# Small Interfering RNAs Targeting a Chromatin-Associated RNA Induce Its Transcriptional Silencing in Human Cells

**DOI:** 10.1128/mcb.00271-22

**Published:** 2022-11-29

**Authors:** Julien Ouvrard, Lisa Muniz, Estelle Nicolas, Didier Trouche

**Affiliations:** a Molecular, Cellular and Developmental Biology Unit, Centre de Biologie Integrative, University of Toulouse, Université Paul Sabatier, CNRS, Toulouse, France; b Equipe labellisée Ligue Contre le Cancer, Toulouse, France

**Keywords:** siRNA, RNA interference, nuclear RNA, transcriptional silencing, noncoding RNA, transcriptional repression

## Abstract

Transcriptional gene silencing by small interfering RNAs (siRNAs) has been widely described in various species, including plants and yeast. In mammals, its extent remains somewhat debated. Previous studies showed that siRNAs targeting gene promoters could induce the silencing of the targeted promoter, although the involvement of off-target mechanisms was also suggested. Here, by using nascent RNA capture and RNA polymerase II chromatin immunoprecipitation, we show that siRNAs targeting a chromatin-associated noncoding RNA induced its transcriptional silencing. Deletion of the sequence targeted by one of these siRNAs on the two alleles by genome editing further showed that this silencing was due to base-pairing of the siRNA to the target. Moreover, by using cells with heterozygous deletion of the target sequence, we showed that only the wild-type allele, but not the deleted allele, was silenced by the siRNA, indicating that transcriptional silencing occurred only in *cis*. Finally, we demonstrated that both Ago1 and Ago2 are involved in this transcriptional silencing. Altogether, our data demonstrate that siRNAs targeting a chromatin-associated RNA at a distance from its promoter induce its transcriptional silencing. Our results thus extend the possible repertoire of endogenous or exogenous interfering RNAs.

## INTRODUCTION

RNA interference (RNAi), first discovered in plants during the 1990s and observed later in other systems, like worms, yeast, and mammals, is a rapid and efficient tool to induce the knockdown of specific transcripts. This main mechanism of posttranscriptional gene silencing has been largely conserved throughout evolution. In mammals and nonmammalian organisms, exogenous or endogenously synthetized small double-stranded RNA molecules of 19 to 24 nucleotides, called small interfering RNA (siRNA), are loaded on the RNA-induced silencing complex (RISC) containing argonaute proteins (Ago) (1). The two strands of siRNAs are then dissociated, and the passenger strand is ejected. The complex is then tethered to target RNAs containing a sequence complementary to the guide strand. When sequence complementarity is high enough, the endoribonuclease activity present in Ago2 cleaves the target RNA between bases 10 and 11 of the siRNA complementary region, leading to the degradation of the target RNA (2, 3).

Given that the above-described processes usually take place in the cytoplasm, RNAi is in this respect a cytoplasmic process functioning at a posttranscriptional level and leading to RNA degradation. Nevertheless, in some organisms, such as yeast or plants, RNAi has been described to work also in the nucleus to silence repeated elements and to nucleate heterochromatin (4). However, the presence of nuclear RNAi in mammals is more controversial. Indeed, initial reports concluded that intronic sequences could not be targeted with RNAi (5, 6). This observation, together with the findings that some RISC proteins were mostly cytoplasmic, led to the conclusion that RNAi is restricted to the cytoplasm in human cells (5). However, in the last 2 decades, various studies have suggested the presence and the action of the RNAi machinery in the nucleus of mammalian cells. Indeed, whereas siRNA loading factors into RISC are exclusively cytoplasmic, other RNAi factors, such as Dicer, Ago1, and Ago2, have been found in the nucleus and can lead to the siRNA-mediated cleavage of chromatin-associated RNAs (7–9). Moreover, the preferential nuclear localization of Ago2 has been demonstrated in specific human tissues (10). It was more recently found that Ago-microRNA (miRNA) complexes can target and silence posttranscriptionally thousands of pre-mRNAs in the nucleus in mouse embryonic stem cells that contain high levels of nuclear Ago proteins (11, 12), although it is still unclear whether all these genes are directly targeted by Ago proteins. Cotranscriptional processes such as alternative splicing can also be affected by siRNA targeting a specific splice site of pre-mRNA (13). Moreover, siRNAs have been successfully used to decrease the expression of nuclear ncRNAs by several research teams (7, 14), including ours (15–17).

In addition, transcriptional gene silencing by siRNAs has also been reported in mammalian cells. siRNAs directed against the promoter of the elongation factor 1α gene repress the expression of a reporter gene driven by this promoter (18). Further studies confirmed for other genes that siRNAs can repress transcription when targeting promoters (19–26) or first exons (14). This was also extended to miRNAs or siRNAs targeting downstream regions of protein-coding genes (27, 28). Transcriptional gene silencing requires an RNA template (29, 30), such as promoter-associated antisense transcripts (31) or noncoding RNAs (ncRNAs) overlapping the gene promoter or downstream regions (26, 32) and involves either Ago1 or Ago2 (19, 32, 33). The targeting of the siRNA to promoters leads to the deposition of repressive chromatin marks at these promoters, such as H3K9me3 and H3K27me3 (9, 21, 25, 33–35) or CpG methylation (20, 23), resulting in transcriptional gene repression. Note that it was proposed that the previously reported transcriptional repression by siRNAs of the vascular endothelial growth factor gene promoter and HIV long termina repeat (LTR) could be due to off-target effects, since a similar effect was observed on reporter systems with a deleted or mutated target site (36). For the HIV LTR, a more recent study showed that siRNAs targeting HIV-1 LTR were specific to the HIV-1 LTR and did not affect the highly related HIV-2 LTR or a panel of other genes regulated in a similar way, arguing against purely off-target effects (37).

Despite these studies, to our knowledge, siRNAs repressing promoters while targeting RNAs far downstream of their promoter region have not been reported so far. Here, we show that siRNAs directed against a chromatin-associated RNA from the very long intergenic noncoding RNA (vlincRNA) family can silence its promoter even when targeted sequences are located thousands of bases downstream from the promoter. Moreover, through the use of genome editing, we found that this effect occurred in *cis* and was not due to off-target mechanisms, thus demonstrating RNA interference mechanisms acting at distance in mammals.

## RESULTS

### siRNAs can efficiently decrease expression of VINK nuclear RNA.

**FIG 1:**
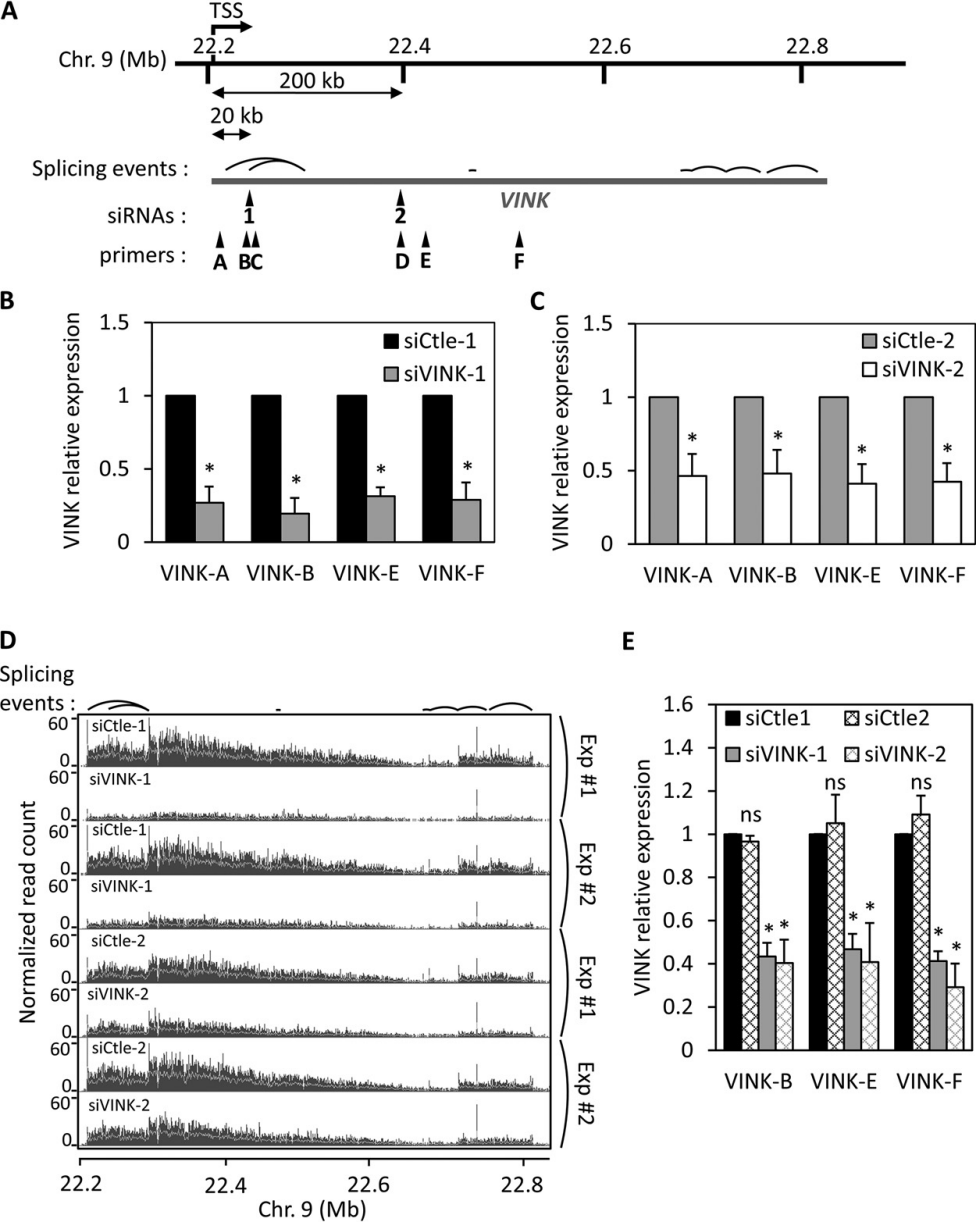
Small interfering RNAs can efficiently decrease VINK expression. (A) Schematic representation of VINK genomic locus, with localization of siRNA binding sites, qPCR primers, and main splicing events detected in RNA-Seq experiments. The exact locations of siRNAs, primers, and main splicing events relative to the TSS of VINK, estimated from RNA-Seq read coverage (unpublished data), are indicated in Tables 2, 3, and 1, respectively. (B) Senescent WI38-RAF1 cells were transfected with VINK-1 or control (Ctle-1) siRNAs. At 72 h later, total RNA was prepared and analyzed by RT-qPCR for the expression of GAPDH mRNA or VINK (using the indicated primers). The amount of VINK RNA was standardized to GAPDH mRNA and calculated relative to a value of 1 for cells transfected with the Ctle-1 siRNA. The means and standard deviations from 5 independent experiments are shown. *, *P* < 0.05 compared to control. (C) Same experiment as in panel B, except that siVINK-2 and another control siRNA (Ctle-2) were used. The means and standard deviations from 7 independent experiments are shown. *, *P* < 0.05 compared to control. (D) WI38-RAF1 cells were treated as for panels B and C, and total RNA was analyzed by RNA-Seq. Two duplicates of RNA-Seq experiments were visualized in the IGB browser, showing the normalized read coverage for the VINK locus. Splicing events consistently detected in RNA-Seq experiments are indicated above the profiles. (E) U2OS cells were transfected with the indicated siRNAs; 48 h later, total RNA was prepared and analyzed by RT-qPCR for the expression of GAPDH mRNA or VINK (using the indicated primers). The amount of VINK RNA was standardized to GAPDH mRNA and calculated relative to a value of 1 for cells transfected with the Ctle-1 siRNA. The means and standard deviations from 3 independent experiments are shown. *, *P* < 0.05 compared to Ctle-1 siRNA; ns, nonsignificant, *P* > 0.1.

In previous works, we successfully used siRNAs to deplete the expression of various RNAs associated with chromatin (15–17). This included depletion of VAD, a senescence-specific ncRNA which belongs to the vlincRNA family (17). More recently, we investigated another vlincRNA also strongly associated with chromatin, with more than 85% present in the chromatin fraction and very weakly spliced, which we named VINK (unpublished data). During the course of this investigation, we designed and transfected siRNAs directed against this vlincRNA in RAF1 oncogene-induced senescent WI38 human cells. By reverse transcription-quantitative PCR (RT-qPCR), we observed that an siRNA against VINK (VINK-1 siRNA) efficiently inhibited VINK expression when measured at various places along the VINK RNA (Fig. 1A and B). A similar result was obtained with a second independent couple of control and VINK siRNAs (VINK-2 siRNA), although VINK inhibition was slightly less efficient with this siRNA (Fig. 1A and C). A dose-response pattern showed that the effect was maximal from 10 nM siRNA (data not shown). The efficient depletion of VINK using each of these two siRNAs was also observed in RNA sequencing (RNA-Seq) experiments (Fig. 1D). Thus, siRNAs against the VINK vlincRNA efficiently repress VINK expression.

From these RNA-Seq data sets, analysis of reads containing at least one spliced junction showed that VINK is indeed poorly spliced, with less than 0.4% of spliced reads relative to all reads aligned in the VINK region. For comparison, we previously showed that the median percentage of spliced reads in annotated genes was around 20% (16). The main splicing junctions are indicated in Fig. 1A and listed in Table 1, along with the locations of primers and siRNA sequences. The most abundant splicing event occurred between a region close to the transcription start site (TSS) and a region around 100 kb downstream (see the sharp peaks in RNA-Seq data sets in Fig. 1D and Table 1).

**TABLE 1:** Main spliced junctions detected by RNA-Seq

Junction	Donor splice site relative to estimated VINK TSS (nt)	Acceptor splice site relative to estimated VINK TSS (nt)	No. of reads containing this junction
1	+330	+84,479	78
2	+21,427	+84,479	16
3	+249,312	+250,728	16
4	+352,560	+352,760	15
5	+457,524	+465,489	36
6	+465,594	+506,188	34
7	+506,311	+550,540	21
8	+551,284	+603,617	10

Data showing that siRNAs can efficiently decrease VINK expression, as well as the expression of other nuclear RNAs, which we have published previously (15–17), were obtained in senescent WI38 cells expressing hTERT, the catalytic subunit of the human telomerase and an estrogen receptor-RAF1 fusion protein. To test whether the depletion of VINK RNA by siRNAs could be observed in other cell types, we made use of the human osteosarcoma U2OS cell line that also expresses VINK. In U2OS cells, VINK is truncated from its 5′ end, the transcript starting around 75 nucleotides (nt) upstream of the region targeted by the siRNA-1 (data not shown). Transfection of each of the two siRNAs against VINK efficiently repressed VINK expression (Fig. 1E).

Thus, altogether these data indicate that siRNAs can efficiently induce the knockdown of the chromatin-associated VINK RNA.

### siRNAs affect transcription of their targeted nuclear RNAs.

**FIG 2:**
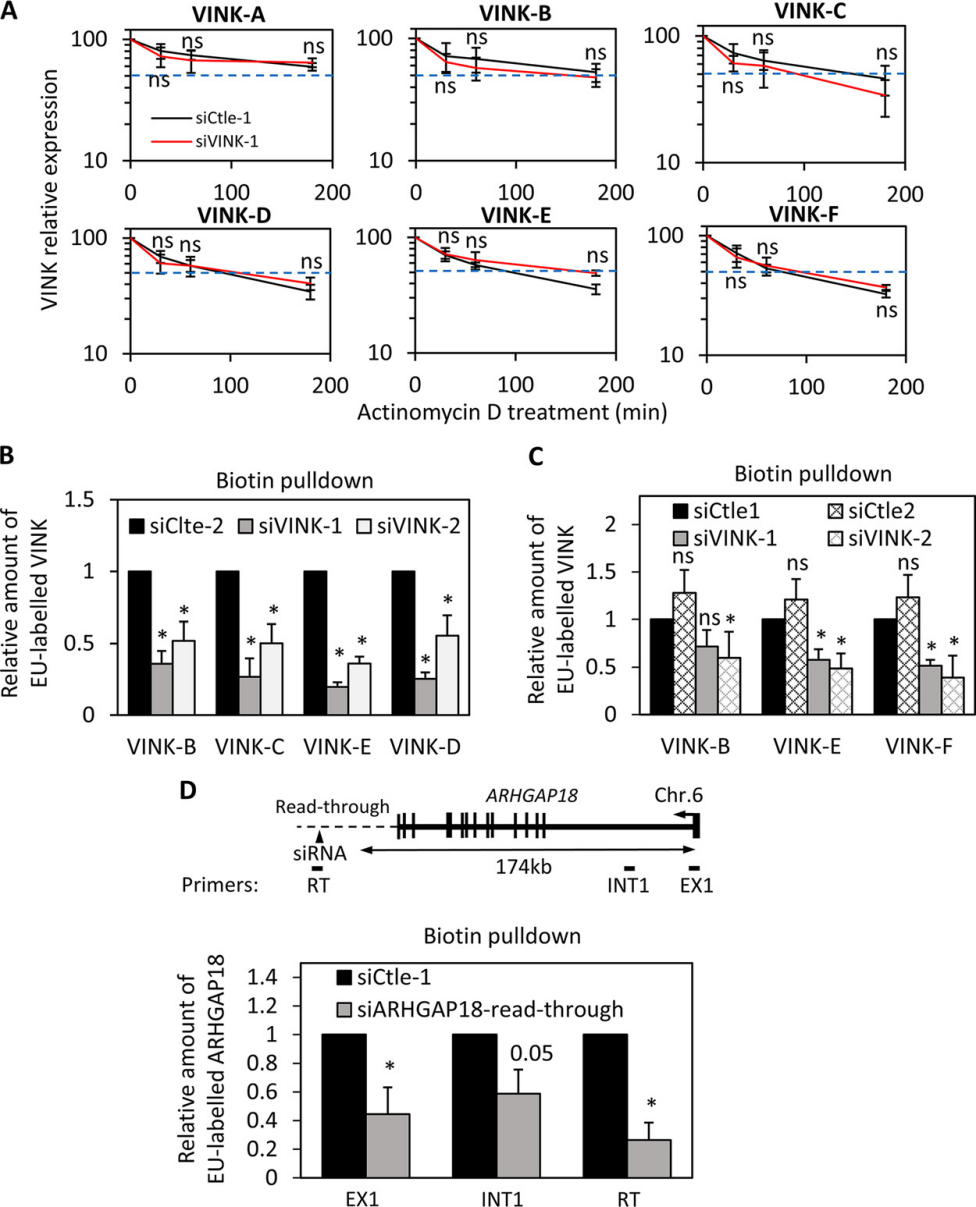
Small interfering RNAs affect VINK transcription. (A) Senescent WI38-RAF1 cells were transfected with the VINK-1 or control (Ctle-1) siRNAs. At 48 h later, actinomycin D (10 μg/mL) was added or not. Cells were harvested after the indicated times. Total RNA was prepared with TRIzol and analyzed by RT-qPCR for the expression of GAPDH mRNA or VINK (using the indicated primers). The amount of VINK RNA was standardized to GAPDH mRNA (which did not vary during this time course of actinomycin D treatment) and calculated relative to a value of 100 for the untreated sample. The means and standard deviations from 3 independent experiments are shown. Student's *t* test was performed to compare VINK1 and Ctle-1 siRNA samples at each time point of actinomycin D treatment. ns (nonsignificant), *P* > 0.1. (B) Senescent WI38-RAF1 cells were transfected with the indicated siRNAs. Nascent RNAs (biotin pulldown) were prepared and analyzed by RT-qPCR for the expression of GAPDH mRNA or VINK (using the indicated primers). The amount of VINK RNA was standardized to GAPDH mRNA and calculated relative to a value of 1 for the Ctle-1 siRNA. The means and standard deviations from 3 independent experiments are shown. *, *P* < 0.05 compared to Ctle-2 siRNA. (C) Same experiment as in panel B, except that U2OS cells were used. *, *P* < 0.05 compared to Ctle-1 siRNA; ns, *P* > 0.1. (D) Senescent WI38-RAF1 cells were transfected with Ctle-1 siRNA or an siRNA directed against ARHGAP18 START RNA (14). Nascent RNAs (biotin pulldown) were prepared and analyzed by RT-qPCR for the expression of GAPDH mRNA or the indicated primers on ARHGAP18. The amount of ARHGAP18 or START RNA was standardized to GAPDH mRNA and calculated relative to a value of 1 for the Ctle-1 siRNA. The means and standard deviations from 3 independent experiments are shown. *, *P* < 0.05 compared to Ctle-1 siRNA; ns, *P* > 0.1; *P* value is shown for the comparison to Ctle-1 siRNA when 0.05 < *P* < 0.1.

Given that the use of siRNAs to target nuclear RNAs is under debate, we intended to investigate the mechanism of this effect. To that goal, we first analyzed whether siRNAs directed against VINK induced its degradation. We transfected senescent WI38 cells with the VINK-1 siRNA (which is the most efficient [Fig. 1B]) or control siRNA and treated transfected cells with actinomycin D to block the transcription. The decrease of VINK expression with time reflected its stability. Using this experimental setting, we found that the half-life of VINK was between 100 and 200 min, depending on the region of VINK analyzed (Fig. 2A). This half-life was longer when we analyzed the 5′ extremity of VINK (VINK-A and VINK-B), certainly because these regions are found in spliced forms of VINK (Fig. 1A and Table 1). However, wherever investigated, VINK half-life was not significantly changed in cells transfected with the VINK-1 siRNA. Moreover, no difference in its half-life VINK degradation was observed upon VINK-1 siRNA transfection following either short or long periods of actinomycin treatment (Fig. 2A and data not shown). Thus, these experiments suggest that the stability of VINK RNA is not affected by the presence of VINK siRNAs.

We next assessed whether VINK siRNAs affected VINK transcription. To that goal, we analyzed ongoing transcription by nascent RNA capture, which relies on metabolic labeling of RNAs being transcribed with a nucleotide analog, allowing their biotinylation and purification on streptavidin beads. Analysis of recovered nascent RNAs thus provides an accurate measure of ongoing transcription. We transfected senescent WI38 cells with each of the two siRNAs against VINK and measured nascent transcripts at different locations along VINK (Fig. 2B). We found that the presence of VINK nascent RNA was strongly decreased upon transfection of the two VINK siRNAs in all regions analyzed (Fig. 2B). Thus, siRNAs targeting VINK RNA decreased its transcription. This effect was also observed in U2OS cells (Fig. 2C), indicating that it is not a specific feature of the WI38 cell line.

To test whether this effect is restricted to VINK or to RNAs from the vlincRNA family, we transfected senescent WI38 cells with an siRNA directed against the ARHGAP18 readthrough RNA belonging to the family of START RNAs that we characterized in a previous study (15). Readthrough RNAs are produced by transcription beyond the transcription termination site and are known to be chromatin associated (38). We also observed that the presence of ARHGAP18 START nascent RNA was strongly decreased upon transfection of the siRNA targeting it (Fig. 2D). ARHGAP18 nascent transcript from which the START RNA originates was also decreased, as measured close to its TSS in its first exon and intron located about 170 kb upstream of the siRNA targeted site (Fig. 2D). These results indicated that the effect of the siRNA directed against ARHGAP18 START RNA was also transcriptional.

Thus, we concluded from these experiments that siRNAs can repress the transcription of the chromatin-associated RNA they target, even when the targeted site is far from the promoter.

### siRNAs targeting VINK RNA influence RNA polymerase II presence at VINK TSS.

**FIG 3:**
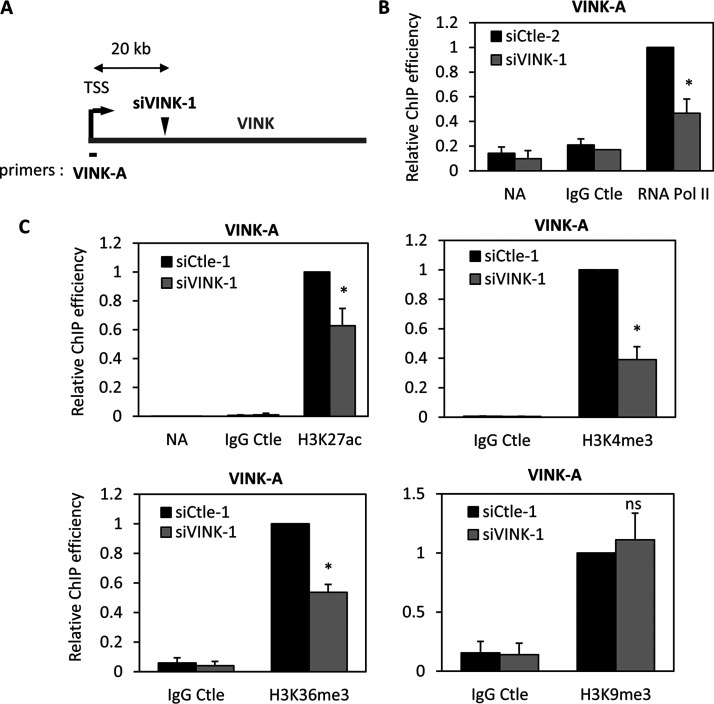
VINK siRNAs affect the presence of RNA polymerase II at VINK TSS. (A) Schematic representation showing the localization of the TSS of VINK and primers used to analyze ChIP experiments. (B) Senescent WI38-RAF1 cells were transfected with the indicated siRNAs (four experiments were performed with Ctle-2 and two with Ctle-1). At 72 h later, fixed chromatin was prepared and subjected to a ChIP experiment using an antibody directed against total RNA Pol II, control IgG, or no antibody as a control, as indicated. The amount of VINK TSS sequence was measured by qPCR with the indicated primers, standardized to the amount of GAPDH TSS sequence, and calculated relative to a value of 1 for the RNA Pol II ChIP from cells transfected by the Ctle siRNA. The means and standard deviations from six independent experiments are shown (four controlled with no antibody [NoAb] and two with a control IgG). *, *P* < 0.05 compared to Ctle siRNA. (C) Same experiment as in panel B, except that anti-H3K27ac, anti-H3K4me3, anti-H3K36me3, and anti-H3K9me3 antibodies were used. The Ctle siRNAs were the siCtle-1 siRNA except for H3K27ac experiments, for which three experiments were performed with Ctle-1 and three with Ctle-2. The amount of VINK TSS sequence was standardized to the amount of GAPDH TSS sequence and to nucleosome occupancy measured by ChIP using an antibody recognizing total histone H3. The result was calculated relative to a value of 1 for relevant ChIPs from cells transfected by the Ctle siRNA. The means and standard deviations from six (H3K27ac) or three (all other antibodies) independent experiments are shown. *, *P* < 0.05 compared to Ctle siRNA.

Inhibition of nascent RNA upon siVINK transfection was observed throughout the VINK transcribed region, including when analyzing regions upstream of the siRNA-targeted sequence (Fig. 1 and 2). siRNA-mediated inhibition of nascent transcription by premature transcription termination was recently observed in Drosophila melanogaster (39). To analyze whether VINK siRNAs induce premature transcription termination or involve the repression of VINK promoter, we analyzed the recruitment of RNA polymerase II (Pol II) to the TSS, which would be unaffected by the induction of premature termination. We thus transfected the VINK-1 or VINK-2 siRNAs and performed chromatin immunoprecipitation (ChIP) using an antibody that recognized total RNA Pol II. We found that both siRNAs against VINK inhibited the presence of RNA Pol II at the VINK TSS compared to that at the control glyceraldehyde-3-phosphate dehydrogenase (GAPDH) TSS (Fig. 3B; data not shown for VINK-2 siRNA). We next analyzed whether this was also associated with the regulation of chromatin marks. We found that the VINK-1 siRNA decreased the presence of the transcription-associated H3K27ac, H3K4me3, and H3K36me3 marks at the VINK TSS, whereas the repressive mark H3K9me3 was unaffected (Fig. 3C). H3K27me3 was undetectable at the VINK TSS, even in the presence of VINK-1 siRNA (data not shown). Thus, these data indicated that siRNAs targeting VINK decrease the presence of transcription-associated chromatin marks and of RNA Pol II at the VINK TSS, thus decreasing VINK transcription. To our knowledge, such an effect on the promoter chromatin landscape induced by when siRNAs target RNAs thousands of bases downstream of their promoter has not been reported before now.

### Repression of transcription by VINK siRNA is dependent on base-pairing to its target site.

We next tested whether the effect of VINK siRNA on VINK transcription was due to base-pairing and not to off-target effects. Indeed, in some instances, transcriptional silencing using siRNAs has been attributed to off-target effects (36).

**FIG 4:**
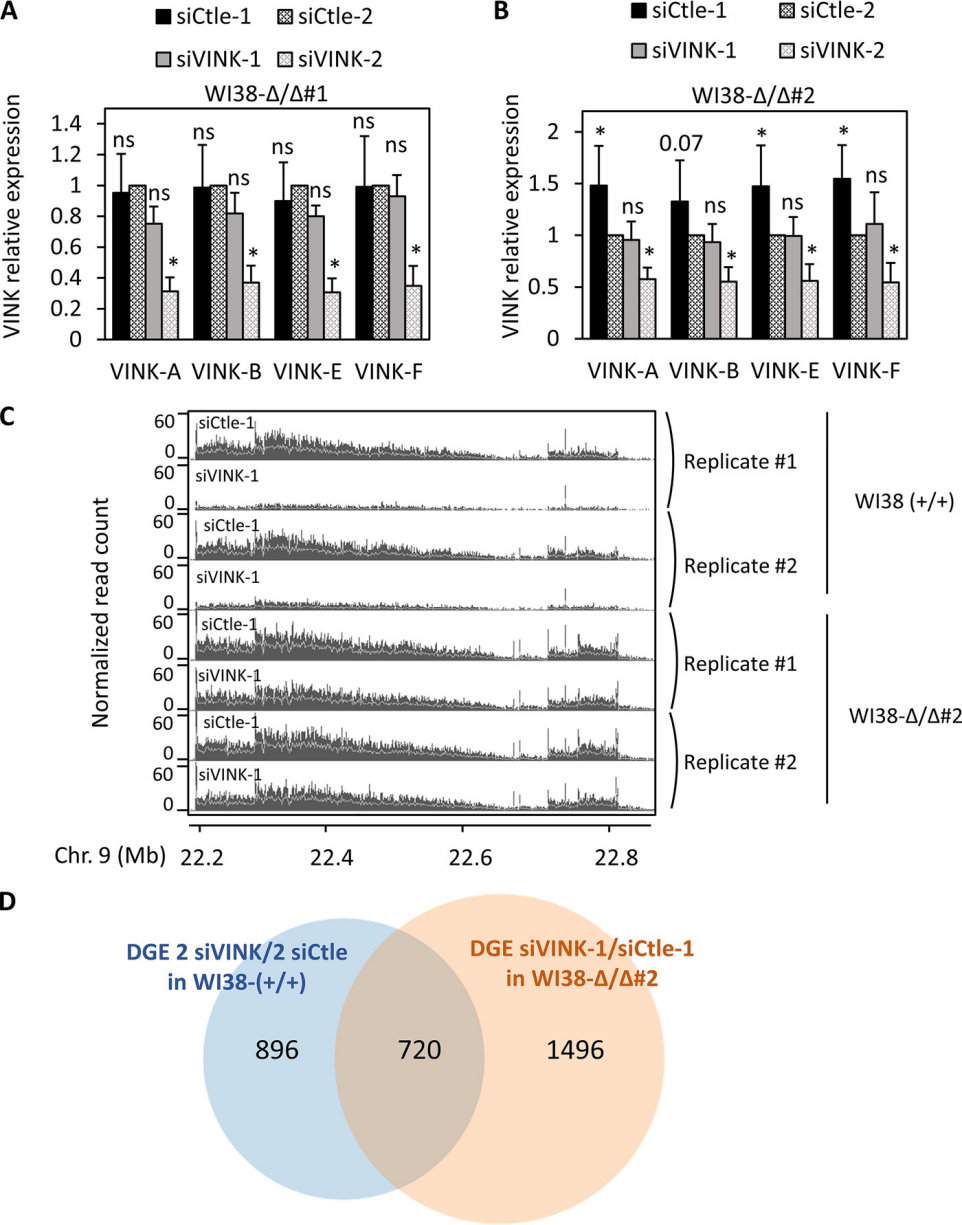
The siRNA target site is required for VINK repression. (A) Senescent WI38-Δ/Δ#1 cells were transfected with the indicated siRNAs. At 72 h later, total RNA was prepared and analyzed by RT-qPCR for the expression of GAPDH mRNA or VINK (using the indicated primers). The amount of VINK RNA was standardized to GAPDH mRNA and calculated relative to a value of 1 for cells transfected with the Ctle-1 siRNA. The means and standard deviations from 3 independent experiments are shown. *, *P* < 0.05 compared to Ctle-2 siRNA; ns (nonsignificant), *P* > 0.1. (B) Same experiment as in panel A, except that senescent WI38-Δ/Δ#2 cells were used. The means and standard deviations from five independent experiments are shown. *, *P* < 0.05 compared to Ctle-2 siRNA; ns, *P* > 0.1; the *P* value is shown for the comparison to Ctle-2 siRNA when 0.05 < *P* < 0.1. Note that siCtle1 led to slightly higher VINK expression than siCtle2 in the cell line, probably due to off-target effects (exemplified in panel D). (C) Senescent parental WI38-RAF1 (WI38-RAF1 WT) or WI38-Δ/Δ#2 cells were transfected with the indicated siRNA, and total RNA was analyzed by RNA-Seq. Two duplicates of RNA-Seq data for the VINK locus are shown. Note that data from WI38-RAF1 wild type were the same as the four upper lines in Fig. 1D. (D) Senescent wild-type WI38 cells were transfected with siVINK-1, siVINK-2, siCtle-1, or siCtle-2, whereas homozygously deleted (Δ/Δ#2) cells were transfected with siVINK-1 or siCtle-1. Total RNAs were prepared from two independent experiments and subjected to RNA-Seq. DGE analysis was performed with the DESeq2 package. A total of 1,616 genes were found significantly deregulated by the two VINK siRNAs compared to the two controls in wild-type cells, and 2,216 genes were significantly deregulated by the siVINK-1 siRNA in the Δ/Δ#2 cell line. These 2,216 genes represented off-target genes, since the siVINK-1 siRNAs did not decrease VINK expression in this cell line (see above). The Venn diagram shows the intersection between these two gene sets. Note that 720 of the 1,616 genes significantly deregulated by the VINK1 and VINK2 siRNAs in wild-type cells were also significantly changed upon VINK-1 siRNA transfection in the deleted cell line.

To analyze whether pairing of the siRNA to its target site is involved, we raised, by genome editing in WI38 cells, a stable cell line that we called WI38-Δ/Δ #1, in which 210 bp encompassing the target site for the VINK-1 siRNA were deleted on the two alleles (data not shown). We next transfected WI38-Δ/Δ #1 cells with the siRNAs directed against *VINK*. Whereas the VINK-2 siRNA worked as in parental cells (compare Fig. 4A and 1C), the VINK-1 siRNA, for which the sequence is not present in WI38-Δ/Δ #1 cells, did not repress VINK expression (Fig. 4A). Importantly, a similar result was also observed in another independent clone deleted for the VINK-1-targeted sequence on the two alleles (WI38-Δ/Δ #2) (Fig. 4B). The absence of effects of the VINK-1 siRNA on VINK expression in WI38-Δ/Δ #2 cells was also obvious from RNA-Seq data that compared the effect of the VINK-1 siRNA in parental WI38 (+/+) and deleted WI38-Δ/Δ #2 cells (Fig. 4C).

Thus, removal of its target sequence abolished the effect of the VINK-1 siRNA on VINK expression, indicating that it represses the expression of its target RNA through base-pairing to its target sequence. Of note, under the conditions we used to achieve VINK knockdown, VINK-1 siRNA induced prominent off-target effects with significant changes in the expression of thousands of genes upon siRNA transfection in the cell line in which the siRNA target site was deleted (Fig. 4D).

### VINK siRNA requires *cis*-targeting to repress transcription of VINK.

Altogether, the above-described data unambiguously demonstrated that the VINK-1 siRNA represses VINK transcription by a process involving base-pairing. This suggested that VINK-1 siRNA targets VINK RNA and induces chromatin modifications at the VINK promoter in *cis* that would lead to the repression of transcriptional initiation. However, we cannot formally rule out the possibility that the VINK-1 siRNA targets and induces the degradation by conventional RNAi mechanisms of a subpopulation of VINK, an effect that would not be observed in our previous experiments because it only represents a minor proportion of VINK. If this population was able to indirectly regulate VINK expression, its depletion could then be signalized to the VINK promoter to repress VINK transcription. Of note, the VINK-1 siRNA targets a region located 300 bases upstream from a donor splice site (see Tables 1 and 2) and may thus target a spliced product of VINK.

**TABLE 2:** List of siRNAs

siRNA name	Sequences^*a*^	Position of 5′ side of siVINK relative to estimated VINK TSS (nt)
siCtle-1	S: GUCAGAGUAUCAUACGUAA-UU AS: P-UUACGUAUGAUACUCUGAC-UU	
siCtle-2	S: GACACAGUUCGAGUACUAA-UU AS: P-UUAGUACUCGAACUGUGUC-UU	
siVINK-1	S: CUUCUCAAGUUUCGAACAA-UU AS: P-UUGUUCGAAACUUGAGAAG-UU	+21,117
siVINK-2	S: GGGAAGGUAAGGAAAGAAA-UU AS: P-UUUCUUUCCUUACCUUCCC-UU	+163,298
siARHGAP18-read_through	S: GGGCAGAGACUGAGACUUU-UU AS: P-AAAGUCUCAGUCUCUGCCC-UU	
siAgo1	S: CCCAGAUACUCCACUAUGA–UU AS: P-UCAUAGUGGAGUAUCUGGG-UU	
siAgo2	S: GGGUAAAGUUUACCAAAGA–UU AS: P-UCUUUGGUAAACUUUACCC-UU	

a S, sense; A, antisense.

**FIG 5:**
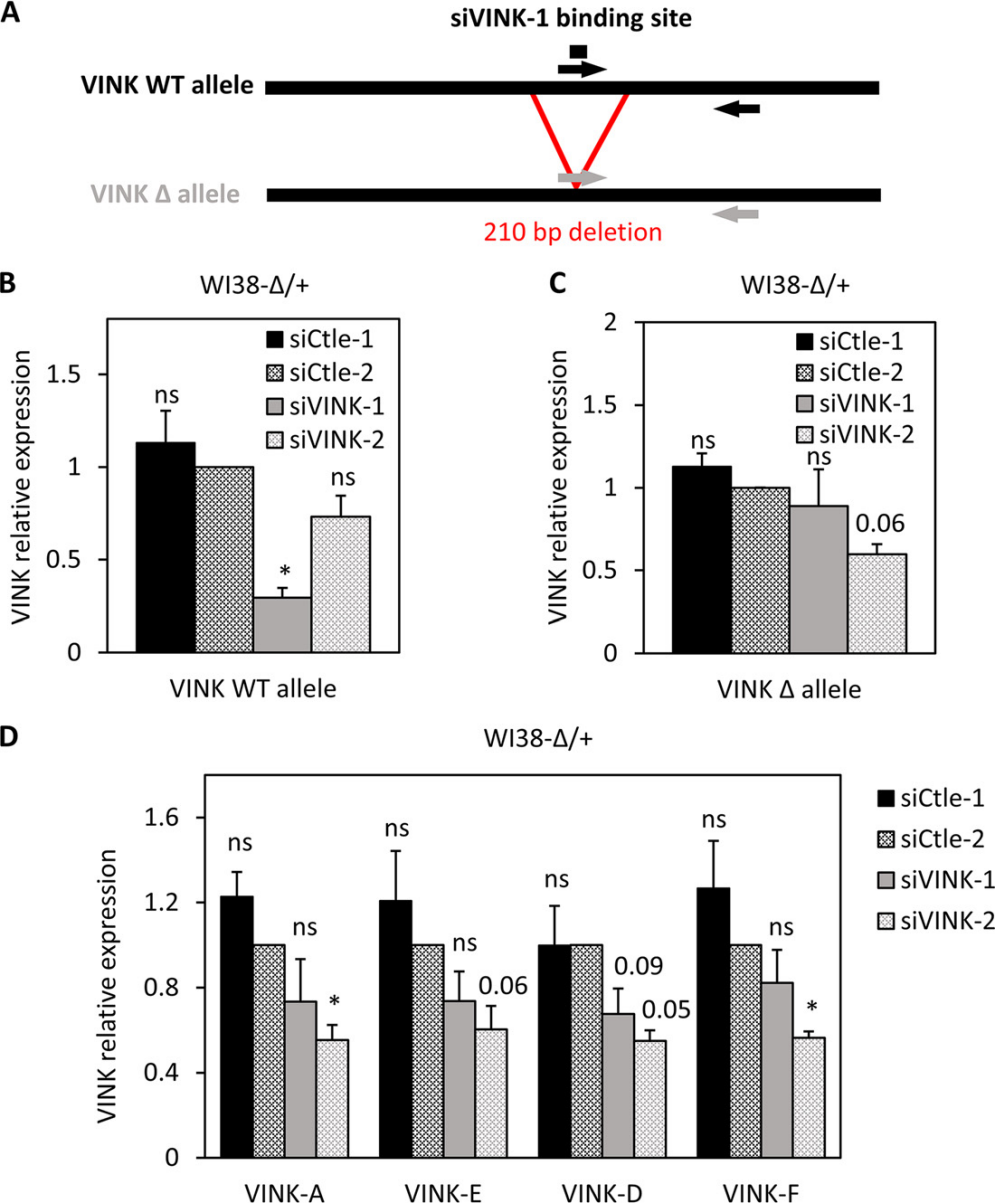
VINK siRNA requires *cis*-targeting to chromatin to repress VINK transcription. (A) Schematic representation of the two alleles of VINK present in WI38-Δ/+ with primers allowing their specific detection. The deleted allele was recognized by a primer located on the deletion junction. (B) Senescent WI38-Δ/+cells were transfected with the indicated siRNAs. At 72 h later, total RNA was prepared and analyzed by RT-qPCR for the expression of GAPDH mRNA or the VINK wild-type allele. The amount of VINK allele was standardized to GAPDH mRNA and calculated relative to a value of 1 for cells transfected with the Ctle-2 siRNA. The means and standard deviations from 3 independent experiments are shown. *, *P* < 0.05 compared to Ctle-2 siRNA; ns, *P* > 0.1. (C) Same experiment as in panel B, except that the deleted VINK allele was analyzed. The means and standard deviations from 3 independent experiments are shown. *, *P* < 0.05 compared to Ctle-2 siRNA; ns, *P* > 0.1; the *P* value is shown for the comparison to Ctle2 siRNA when 0.05 < *P* < 0.1. (D) Same experiment as in panels B and C, except that total VINK expression was analyzed with the indicated primers. The means and standard deviations from 3 independent experiments are shown. *, *P* < 0.05 compared to Ctle-2 siRNA; ns, *P* > 0.1; the *P* value is shown for the comparison to Ctle-2 siRNA when 0.05 < *P* < 0.1.

To formally demonstrate that VINK siRNAs act in *cis*, we raised by genome editing a cell line in which only one of the two alleles encoding VINK could be targeted by the VINK-1 siRNA, the other allele being deleted for the target sequence (which we called WI38-Δ/+). If the effects of siRNAs were due to base-pairing at chromatin-inducing local chromatin modifications, only the siRNA-targeted allele would be repressed. On the contrary, if the repression of VINK transcription was indirect, then the two alleles would be similarly regulated. We thus transfected WI38-Δ/+ cells with VINK-1 and VINK-2 siRNAs and performed RT-qPCR to monitor allele-specific VINK expression (see Fig. 5A for the design of PCR primers). Each of the two siRNAs inhibited the expression of the wild-type allele, with a greater effect of the VINK-1 siRNA than the VINK-2 siRNA (Fig. 5B), as in previous experiments (Fig. 1B to D). By contrast, on the deleted allele, while we still observed repression by the VINK-2 siRNA, repression by the VINK-1 siRNA was abolished (Fig. 5C). The effect of the VINK-1 siRNA on total VINK expression in WI38-Δ/+ was weaker than on the wild-type allele (compare Fig. 5D and B) or than in parental cells (compare Fig. 5D with Fig. 1B), as expected since only one allele was affected.

Altogether, these data thus demonstrate that the effect of VINK-1 siRNA on VINK transcription is mediated in *cis* by base-pairing of the siRNA to its target site on the chromatin-associated VINK RNA.

### Ago1 and Ago2 are involved in siRNA-mediated VINK transcriptional repression.

**FIG 6:**
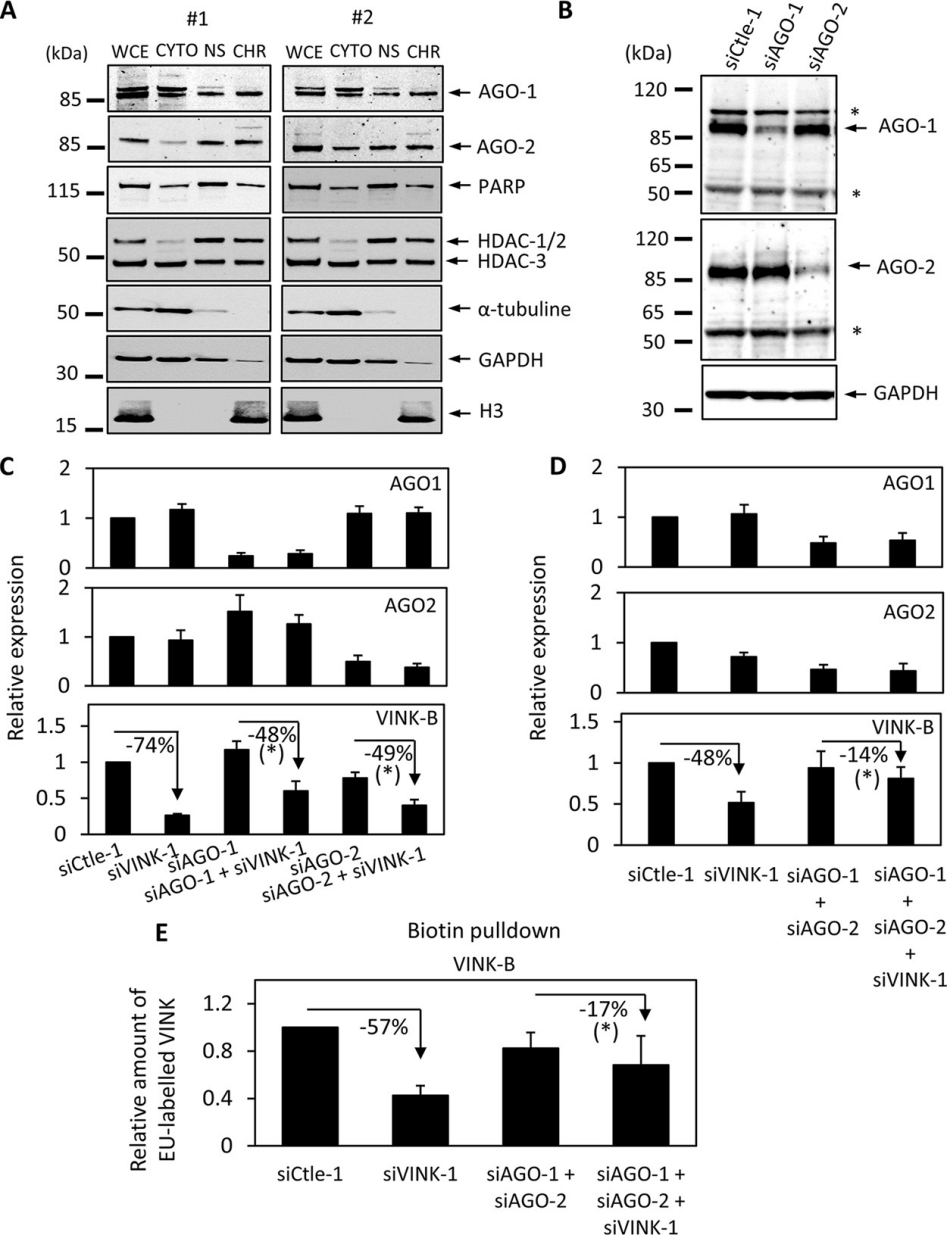
Ago1 and Ago2 are involved in VINK transcriptional repression by VINK siRNA. (A) Senescent WI38 cells were recovered and subjected to fractionation experiments. Whole-cell extracts (WCE) and cytoplasmic (cyto), nuclear soluble (NS), and chromatin (CHR) fractions from the same amount of cells were analyzed by Western blotting using the indicated antibodies. (B) Senescent WI38 cells were transfected with 30 nM of the indicated siRNAs. At 72 h later, cells were harvested and total cell extracts were subjected to Western blot analysis with antibodies directed against Ago1, Ago2, or GAPDH as a loading control. The asterisks indicate nonspecific bands. Identical results were obtained in three independent experiments. (C) Senescent WI38 cells were transfected with 30 nM of the indicated siRNAs. The amount of siRNAs was kept constant at 60 nM, using Ctle-1 siRNA. At 72 h later, total RNA was prepared and analyzed by RT-qPCR for the expression of GAPDH, Ago1, or Ago2 mRNA or VINK. The amount of Ago1, Ago2, or VINK was standardized to GAPDH mRNA and calculated relative to a value of 1 for cells transfected with the Ctle-1 siRNA. The means and standard deviations from 3 independent experiments are shown. *, *P* < 0.05 comparing the effect of siVINK-1 in cells depleted for Ago1 or Ago2 to its effect alone (paired Student's *t* test). (D) Senescent WI38 cells were transfected with 25 nM of the indicated siRNAs. The amount of siRNAs was kept constant at 75 nM using Ctle-1 siRNA. At 72 h later, total RNA was prepared and analyzed by RT-qPCR for the expression of GAPDH, Ago1, or Ago2 mRNA or VINK. The amount of Ago1 or Ago2 mRNA or VINK was standardized to GAPDH mRNA and calculated relative to a value of 1 for cells transfected with the Ctle-1 siRNA. The means and standard deviations from 6 independent experiments are shown. *, *P* < 0.05 when comparing the effect of siVINK-1 in cells depleted for Ago1 and Ago2 to its effect alone (paired Student's *t* test). (E) Same experiment as in panel D, except that nascent RNAs were recovered. The amount of VINK was standardized to GAPDH and calculated relative to a value of 1 for cells transfected with the Ctle-1 siRNA. The means and standard deviations from three independent experiments are shown. *, *P* < 0.05 when comparing the effect of siVINK-1 in cells depleted for Ago1 and Ago2 to its effect alone (paired Student's *t* test).

It has been shown that transcriptional silencing by siRNA targeting promoters can involve either Ago1 or Ago2 (19, 32, 33). We thus investigated whether these proteins also participated in the transcription repression by siRNAs targeting VINK. We first tested whether Ago1 or Ago2 could be associated with chromatin in WI38 cells, since the nuclear localization of Ago proteins is somewhat debatable. To that goal, we performed cell fractionation experiments to obtain cytoplasmic, nuclear soluble, and chromatin protein fractions (Fig. 6A). Cell fractionation was effective, since α-tubulin and GAPDH were mostly found in the cytoplasmic fraction, poly(ADP-ribose) polymerase (PARP) and histone deacetylase 1 and 2 (HDAC1/2) were found in the nuclear soluble fraction, and histone H3 was in the chromatin fraction. Interestingly, both Ago1 and Ago2 were detected in the three fractions, with an important amount present with chromatin. Comparison with the GAPDH or α-tubulin profile indicated that this amount was unlikely due to contamination of the chromatin fraction with cytoplasmic proteins, and thus it indicated that Ago1 and Ago2 are present at the chromatin in significant amounts. To test whether Ago1 or Ago2 expression was required for transcriptional repression by VINK1 siRNA, we made use of siRNAs targeting either Ago1 or Ago2. These two siRNAs were efficient and specific, as observed by Western blotting (Fig. 6B). We thus tested the effect of Ago1 and/or Ago2 knockdown on VINK repression by the VINK-1 siRNA. We found that siRNAs against Ago1 and Ago2 had similar efficiencies on their respective target whether or not they were transfected together with control or VINK-1 siRNAs, indicating that transfection of many siRNAs together did not affect their efficiency (Fig. 6C and D). In contrast, the effect of the VINK-1 siRNA was weaker in the presence of either Ago1 or Ago2 siRNA (Fig. 6C) and was nearly abolished when we knocked down both Ago1 and Ago2 (Fig. 6D). Importantly, this last result was also observed when analyzing nascent RNA expression, confirming that it occurs at the transcriptional level (Fig. 6E). Thus, these results indicate that both Ago1 and Ago2 are involved in siRNA-mediated transcriptional repression of VINK.

## DISCUSSION

In this study, we demonstrated that siRNAs can induce efficient and specific transcriptional silencing of an RNA associated with chromatin. We observed the silencing of the VINK promoter by using siRNAs targeting the RNA in regions located thousands of bases downstream of the promoter, a phenomenon that, to our knowledge, has never been described in mammals. We further demonstrated the involvement of Ago1 and Ago2 in this process. This silencing was lost when the sequences targeted by the siRNA were deleted, ruling out off-target effects and demonstrating that base-pairing to its target sequence is required. Note that we cannot formally conclude whether this base-pairing occurs on VINK RNA or on its transcribed template DNA.

The first question raised by our finding is whether the interference we have shown here with exogenously delivered siRNAs can also take place under natural conditions with endogenous siRNAs. Indeed, in various species, RNAi mechanisms have been shown to be involved in transcriptional silencing occurring at specific chromatin domains, such as pericentric heterochromatin in yeast (4). Moreover, the involvement of endogenous antisense RNAs in the establishment or maintenance of heterochromatin silencing is well-established (40, 35). Interestingly, it was recently proposed that tRNA-derived small RNAs could be endogenous regulators of many genes, albeit by a different interference mechanism requiring cleavage by Ago2 (12), and other examples of endogenous siRNAs produced from protein-coding gene bodies have been described (41). Thus, we can speculate that endogenous RNAs complementary to chromatin-associated RNAs could be important regulators of the expression of these RNAs in mammals by triggering RNAi and transcriptional gene silencing.

To our knowledge, our study is the first to show that interfering RNAs targeting an RNA in regions far downstream of a promoter can induce its transcriptional silencing in mammals. Such long-distance effects have been widely described in other species, in particular in plants. In these cases, it relies on RNA-dependent RNA polymerase (RdRP), which mediates amplification and propagation of double-strand RNAs toward the 5′ end of RNAs (4). However, in mammals, RdRP is not conserved. What could thus be the mechanism involved in the process we describe here? It is known that double-strand RNAs targeting the vicinity of a promoter (upstream promoter or the first exon) can lead to transcriptional silencing of the promoter through the induction of repressive epigenetic marks in a manner dependent on some RISC components (20, 23, 25). Here, we can speculate that the silencing of the promoter at a distance from the siRNA target site could be achieved by chromatin folding that would bring the VINK-siRNA hybrid and the promoter in close proximity. As a consequence, this would change the chromatin landscape at the promoters, as proposed for miRNAs targeting the 3′ ends of genes (27, 28). Of note, the siVINK-1 siRNA used in our studies targets a sequence close to an internal promoter, which is active in U2OS cells (data not shown) and probably also in WI38 (unpublished data). This second VINK TSS could favor its spatial proximity with the VINK promoter located more than 30 kb upstream. However, the fact that we observed transcriptional silencing with many different siRNAs argues against the importance of specific preexisting chromatin folding that would drive this silencing.

Contrary to the studies showing transcriptional gene silencing by miRNA targeting downstream regions of genes (27, 28), we observed a decrease in the presence of marks associated with transcription, rather than an increase in repressive marks. Our data suggested the involvement of Ago proteins in this silencing. First, mere steric blocking by the VINK-siRNA hybrid is probably not sufficient for this silencing, since an antisense oligonucleotide targeting the same sequence as the VINK-1 siRNA does not induce transcriptional silencing, despite readily decreasing VINK expression (data not shown). Moreover, we found that Ago1 and Ago2 protein depletion partially reversed VINK repression and that significant levels of Ago1 and Ago2 proteins were present at the chromatin in WI38 cells. However, by ChIP, we were not able to detect binding of Ago1 or Ago2 to the site targeted by the siRNA. We thus cannot formally rule out the possibility that the requirement of Ago proteins is indirect. Along this line, it would be interesting to analyze the relative importance of the siRNA seed domain versus other parts of the siRNA and whether 100% complementarity is required for silencing.

Despite the fact that intronic sequences are also found associated with chromatin, siRNAs directed against intronic sequences of pre-mRNAs are usually not effective in decreasing the expression of pre-mRNAs (5, 6, 42). This finding first indicates that transcription of the target sequence is not sufficient to induce transcriptional silencing by siRNA, suggesting that the target of the siRNA is chromatin-associated RNA and not the DNA being transcribed. Splicing occurs cotranscriptionally, leading to the degradation of introns, which thus greatly decreases the window of opportunity for the siRNA to bind efficiently to its target RNA or to induce chromatin modifications when targeted. Moreover, siRNAs directed against a lncRNA did not induce transcriptional silencing, except when targeting the first exon (14). Thus, whatever the mechanism involved, it does not operate on any RNA associated with chromatin and we can speculate that the difference lies in the time of residence of the targeted RNA at chromatin.

The use of siRNAs to silence nuclear RNAs has been debated for a long time, because of the low levels of some RNAi proteins in the nucleus. As a consequence, siRNA-mediated degradation of RNA is mostly efficient in the cytoplasm. Nonetheless, as discussed above, siRNAs targeting promoters can in some instances repress transcription from the promoter they target. Here, we showed that the promoter of chromatin-associated RNAs can be silenced using siRNAs targeting these RNAs in regions located far downstream of the promoter. Thus, siRNAs can represent interesting tools to investigate the function of chromatin-associated RNA by allowing their efficient and specific knockdown, even if the promoter is not well-characterized or if the RNA is produced through multiple promoters. In addition, and coupled to a method allowing the degradation of RNA without interfering with any transcription step, it may help to distinguish between the role of the RNA itself and the role of its transcription, an often-raised question in the field. Most importantly, since we found that siRNAs inhibit the transcription of the allele they target but not of the allele they do not target, they could allow studying *cis* effects of nuclear noncoding RNAs or of their transcription, provided that an existing polymorphism or heterozygous genome editing, as we have done here, allows targeting a single allele.

Note, however, that under the conditions we used to achieve the knockdown of VINK, VINK-1 siRNA induced prominent off-target effects with significant changes in the expression of thousands of genes upon siRNA transfection in the cell line in which the siRNA target site was deleted. Moreover, the use of two independent siRNAs was not sufficient to cope with off-target effects. Indeed, most of the genes showing expression changes with two independent siRNAs directed against VINK in wild-type cells had a tendency to be changed in a similar way in the cell line deleted for the siRNA-1 target site (data not shown). These off-target effects were probably due to the fact that an siRNA can bind to and degrade target RNAs without perfect sequence complementarity, as the seed region of only 6 to 7 nucleotides in the siRNA is involved in targeting numerous RNAs (43). It is possible that they are exacerbated when siRNAs do not have any target in the cytoplasm, such as for siRNAs directed against chromatin-associated RNAs or control siRNAs. Indeed, we also found thousands of differentially expressed genes (DEG) between two different control siRNAs (data not shown). Importantly, we have not been able to dissociate the base-pairing-dependent repression of VINK expression to off-target effects by changing the amount of siRNAs or the time after transfection at which we harvest cells (data not shown). It will thus be critically important for functional studies to include an experimental strategy ruling out off-target effects, such as overexpression of an siRNA-resistant form of the targeted RNA or, as we performed here, removal of the siRNA target site by genome editing. When possible, this latter strategy is probably better, since it maintains the normal production of the RNA at the normal place in the genome and in the nucleus, which is sometimes critical for the function of noncoding RNAs.

Finally, our findings suggest that siRNAs targeting chromatin-associated RNAs may have therapeutic potential. Indeed, the therapeutic value of siRNAs is currently being widely investigated (44). Moreover, the relation of chromatin-associated RNAs with cancer progression has been documented in many instances, such as PVT1, H19, MALAT1, and HOTAIR (for a recent review, see reference 45). RNAs from the vlincRNA family are also overexpressed in cancer cells (46), and this overexpression may participate in allowing these cells to survive genotoxic stress (47). Targeting these RNAs with siRNAs may thus have therapeutic potential. Moreover, by modifying the chromatin landscape at their promoters and thus potentially reversing epigenetic information, it may have long-term benefits.

## MATERIALS AND METHODS

### Cell culture.

WI38-hTERT/ER-RAF1 immortalized fibroblastic cell line was kindly provided by C. Mann (48). They were grown in minimal essential medium supplemented with l-glutamine, nonessential amino acids, sodium pyruvate, penicillin-streptomycin, and 10% fetal bovine serum under 5% CO_2_ and 5% O_2_. Senescence was induced by treating cells with 20 nM 4-hydroxy-tamoxifen for 3 days. U2OS cells (ATCC) were grown in Dulbecco’s modified Eagle’s medium containing Glutamax supplemented with sodium pyruvate, penicillin-streptomycin, and 10% fetal bovine serum under 5% CO_2_. WI38 cells and derivatives were transfected using the siRNA transfection reagent Dharmafect 4 (Dharmacon) following the manufacturer’s instructions, with 100 nM siRNA (unless indicated in a figure legend), and harvested 72 h later. U2OS cells were transfected with the INTERFERin transfection reagent (Polyplus) or with Dharmafect 4 according to the manufacturers’ instructions with 50 nM siRNA and harvested 48 h later. For RNA stability analysis, actinomycin D (Sigma) was added at 10 μg/mL. The siRNAs used are listed in Table 2.

### Cell fractionation, Western blotting, and antibodies.

Phosphatases and protease inhibitors (ThermoFisher catalog number 78441) were added in all buffers. Fresh trypsinized cells were lysed in 5 cell volumes of lysis buffer (10 mM Tris [pH 8], 10 mM NaCl, 2 mM MgCl_2_). After 5 min on ice, 0.5% NP-40 was added. After another 10 min on ice, one-third of the volume was kept as whole-cell extract, and the remaining lysate was centrifuged for 10 min at 5,000 rpm. The supernatant corresponded to the cytosolic fraction. The pellet (nuclei) was resuspended in 1 volume of buffer 3 (20 mM HEPES [pH 7.9], 420 mM NaCl, 1.5 mM MgCl_2_, 10% glycerol) and then incubated for 30 min on ice, with shaking every 5 min. After 10 min of centrifugation at maximum speed, the supernatant was kept as the nuclear soluble fraction. The pellet was the chromatin fraction. All the fractions (cytosol, nuclear soluble, and chromatin) were equilibrated to the same final volume and same buffer compositions, adding 1% SDS in each. The whole-cell extract was also adjusted to the same composition. The whole-cell extract and chromatin were sonicated until viscosity disappeared. Laemmli sample buffer was added to the samples, and the same volume of each fraction was loaded on an SDS-PAGE, except for the whole-cell extract, for which twice the volume of fractions was loaded.

Western blotting was performed using standard protocols. Ago1 and Ago2 were detected using the monoclonal anti-AGO1 (6H1L4) antibody from Invitrogen (used at 1/1,000) and the monoclonal anti-AGO2 (R.386.2) antibody from Invitrogen (1/1,000); GAPDH was detected using the monoclonal anti-GAPDH (MAB374) antibody from Millipore (1/10,000); PARP was detected with anti-PARP from Cell Signaling (catalog number 9542) at 1/1,000; HDAC1, HDAC2, and HDAC3 were detected with anti-HDAC-3 from BD Transduction Lab (catalog number 611125) at 1/500; H3 was detected with anti-H3 from Abcam (Ab1791) at 1/1,000; and α-tubulin was detected with anti-tubulin from Sigma (T6199) at 1/1,000.

For the ChIP experiment, anti-RNA Pol II was from Bethyl Laboratories (A304-405A), anti-H3K27ac was from Abcam (Ab4729), anti-H3K4me3 was from Diagenode (pAb-003), anti-H3K9me3 was from Abcam (Ab8898), anti-H3 was from Abcam (Ab1791), and anti-H3K36me3 was from Abcam (Ab9050). Purified rabbit IgG control was from Millipore (PP64B).

### Genome editing.

Genome editing of WI38 was performed with CRISPR-Cas9 tools in ribonucleic particle (RNP) format. Briefly, the Alt-R S.p. HiFi Cas9 nuclease V3 (Integrated DNA Technologies [IDT]) was assembled with an equimolar amount of hybridized single guide RNA (tracrRNA and crRNA from IDT) to generate RNPs. According to IDT recommendations, RNPs were transfected at 5 nM each with Lipofectamine CRISPRMAX (Thermofisher). Cells were harvested 1 week later to analyze the efficiency of genome editing. Genomic DNA was extracted with a Master Pure purification kit (Epicentre) according to the manufacturer’s instructions, except that 250 μg of proteinase K was used for cell lysis. Genome editing efficiency was checked by PCR on genomic DNA from the transfected population with the GoTaq G2 polymerase (Promega) using primers surrounding the 210-bp deleted region. PCR products were visualized on an agarose gel. Clones were then isolated and screened by PCR as described above. Two homozygous clones and one heterozygous clone were selected. The sequences of sgRNAs were the following: sgRNA-1, TAGGAGGCCAGTGCTCCAGG; sgRNA-2, TTCCTGACTCTTGAAAACCA.

### RNA extraction, reverse transcription, and qPCR.

Except when indicated, total RNA was prepared using the MasterPure RNA purification kit (Epicentre) according to the manufacturer’s instructions, except that 250 μg of proteinase K was used for cell lysis. For the experiment shown in Fig. 2A, TRIzol (Sigma) was used. Briefly, adherent cells were directly lysed with 0.1 mL/cm^2^ TRIzol during 10 min, and the lysate was harvested before adding 0.1 mL of chloroform/mL of TRIzol. After centrifugation, the upper phase was collected and the Epicentre kit was used for the DNase digestion and subsequent precipitation. After total nucleic acids recovery, DNA was removed with a cocktail of DNase I and Baseline Zero DNase supplemented with Riboguard RNase inhibitor for 45 min at 37°C.

A 500-ng amount of total RNA was reverse transcribed with SuperScript III reverse transcriptase (Invitrogen) according to the manufacturer’s instructions. A condition without SuperScript III was included for each sample and analyzed by qPCR using GAPDH exon 9 primers to check for DNA contamination. qPCR was performed in triplicate with TB green Premix *Ex Taq* from TaKaRa and the Bio-Rad CFX thermocycler. GADPH mRNA expression was used to normalize the results of RT-qPCR. Primers are listed in Table 3.

**TABLE 3:** List of primers

Primer name	Sequences^*a*^	Position of the amplicon relative to estimated VINK TSS (nt)
VINK-A	FW: CTGGACACACCCTAGGCAAG RV: ATCCCTGTTGGTTATTCACAGC	+207 to +323
VINK-B	FW: CCACAACCCCCATCTCTCTG RV: CCCATCTTTGGTTCTTCTGTC	+21,840 to +21,390
VINK-C	FW: TGGGTAGCATCTTCATCGGTG RV: GAAGTGAGGGGAGTAGACAAG	+22,497 to +22,751
VINK-D	FW: ACGCATGGGTGCCCTGTTTG RV: ATAGATCTTGGAGGGTGCCAG	+162,326 to +162,445
VINK-E	FW: ACCACTATACAATTGCAGCAGG RV: CTGTCTGTTTAGGTACCTGGC	+199,375 to +199,539
VINK-F	FW: GAGTGTCATCAGTAGTCTTAGCC RV: CGAGGTCCAATATCCCTAACC	+296,406 to +296,540
420 bp encompassing the deletion product PCR primers	FW: GCATGAGTCATCTGGCTGTG RV: CCCATCTTTGGTTCTTCTGTC	
WT VINK allele primers	FW: CCACAACCCCCATCTCTCTG RV: CCCATCTTTGGTTCTTCTGTC	
Δ VINK allele primers	FW: ATATAGGAGGCCAGTGCTCCCCATG RV: CCCATCTTTGGTTCTTCTGTC	
GAPDH exon 9 primers	FW: TGACAACGAATTTGGCTACAGC RV: CTCTTCCTCTTGTGCTCTTGC	
GAPDH promoter primers	FW: AAATTGAGCCCGCAGCCTCC RV: GCGACGCAAAAGAAGATGCG	
ARHGAP18-EX1	FW: CAGGCGAGACAGGAACTTCTTT RV: CTGCCTTTGCATGGCTGTTC	
ARHGAP18-INT1	FW: GCTGCTGGAGTGAAATGTGG RV: TGTTCAGCTAGTGAGAAGGTC	
ARHGAP18-read_through	FW: GTTCCTAGAGATTGATCTGAGG RV: GAATAGACTTGGGTTGCCACG	
AGO1	FW: CAGTGGACACCAACATCACC RV: AAACGGTTGTCATCCCAAAG	
AGO2	FW: CGCGTCCGAAGGCTGCTCTA RV: TGGCTGTGCCTTGTAAAACGCT	

a FW, forward; RV, reverse.

### Nascent RNA capture.

Cells were incubated with 0.2 mM ethynyl-uridine, a uridine analog, for 1 h. Cells were then harvested, total RNA was prepared, and nascent RNA was purified from 2 μg of total RNA with the Click IT nascent RNA capture kit from Life Technologies according to the manufacturer’s recommendations.

### ChIP.

ChIP was performed on 10 × 10^6^ transfected senescent WI38 cells as previously described (17), except that the nuclear lysis buffer was diluted twice before use and sonicated nuclei were diluted 5 times in the dilution buffer. A total of 10 million cells transfected with siRNA were cross-linked for 15 min using 1% formaldehyde directly in the culture medium. Then, 0.125 M glycine was then added for 5 min. After two washes with phosphate-buffered saline, cells were scraped and frozen at −80°C. Cells were lysed with 3 mL of lysis buffer [5 mM piperazine-*N*,*N*′-bis(2-ethanesulfonic acid) (pH 8), 85 mM KCl, 0.5% NP-40] and homogenized 40 times with a dounce tool (20 times, pause for 2 min, 20 more times). After centrifugation, nuclear pellets were resuspended in 1.5 mL of nuclear lysis buffer (25 mM Tris [pH 8.1], 5 mM EDTA, 0.5% SDS) and sonicated 10 times for 5 s (power setting 0.5 on, 0.5 off and 50% amplitude; Branson Sonifier 250) to obtain DNA fragments of about 500 bp. DNA concentration was determined using a Nanodrop system, and samples were adjusted to the same concentration of chromatin. Samples were diluted five times in dilution buffer (0.01% SDS, 1.1% Triton X-100, 1.2 mM EDTA [pH 8], 17 mM Tris [pH 8.1], 167 mM NaCl) and precleared for 2 h with 250 μL of previously blocked 50% protein A and protein G beads (Sigma P-7786 and P-3296, respectively). Blocking was achieved by incubating the beads with 0.5 mg/mL of Ultrapure bovine serum albumin and 0.2 mg/mL of salmon sperm DNA for 4 h at 4°C. A 100-μL volume of chromatin was kept for inputs. A 100-μg amount of precleared samples per ChIP was incubated overnight for RNA Pol II ChIP or a 40-μg amount for histones ChIP with 4 μg of antibody at 4°C. A mock sample without antibody was processed similarly. Then, 40 μL of blocked 50% protein A/G beads was added for 2 h at 4°C to recover immune complexes. Beads were washed once in dialysis buffer (2 mM EDTA, 50 mM Tris [pH 8], 0.2% Sarkosyl), five times in wash buffer (100 mM Tris [pH 8.8], 500 mM LiCl, 1% NP-40, 1% sodium deoxycholate) and twice in TE buffer (10 mM Tris [pH 8], 1 mM EDTA). The bead-chromatin complexes were resuspended in 200 μL of TE buffer and incubated 30 min at 37°C with 10 μg of RNase A (Abcam), as well as input DNA. Formaldehyde cross-linking was reversed in the presence of 0.2% SDS at 70°C overnight with shaking. After 2 h of proteinase K (0.2 mg/mL) treatment at 45°C, immunoprecipitated and input DNA were purified on columns using an Illustra GFX kit (GE Healthcare). All buffers for ChIP experiments were supplemented with EDTA-free protease inhibitor cocktail (Roche) and filtered at 0.2 μm. Results were analyzed by qPCR. Primers are listed in Table 3.

### Statistics.

In all figures with statistical analysis results, data were normalized to 1 relative to a control included in each experiment (usually a control siRNA). For significance analysis, the log_2_ fold change compared to this control was calculated in order to obtain data with a normal distribution. All experiments were then pooled together to calculate the variance of these experiments with a sufficient number of points. We then determined using Student's *t* test with this fixed variance the probability that the mean of each data set was equal to 0 (the value of the control), which represented the *P* value of the difference from the control. In some cases, we using the paired Student's *t* test to determine the probability that the mean of two populations was equal, which represented the *P* value of the difference between the two populations. Asterisks are included in the figures when the *P* value was <0.05. The *P* values are indicated in the figure when they were between 0.05 and 0.1. When above 0.1, the difference was considered nonsignificant.

### RNA-Seq.

We performed strand-specific RNA-Seq, relying on UTP incorporation in the second cDNA strand. For each sample, 5 to 10 μg of total RNA was submitted to EMBL-GeneCore, Heidelberg, Germany. Paired-end sequencing was performed by using Illumina’s NextSeq 500 technology. Two replicates of each sample were sequenced.

The quality of each raw sequencing file (fastq) was verified with FastQC (49). Files were aligned to the reference human genome (hg38) in paired-end mode with STAR version 2.5.2b and processed (sorting and indexing) with samtools (50). rDNA contamination was removed in the alignment process. Uniquely aligned reads were counted, per gene_id, using HT-seq version 0.6.1 in a strand-specific mode with the union count method parameter (51). The features’ annotation used for counts was constructed from NCBI refseq annotation gtf file from UCSC, taking the entire locus of reference for each gene. After removing low-expressed counts (>4 reads over the 4 samples), differential analysis was performed with DESeq2 Bioconductor R package, version 1.22.1 (52). Counts were normalized using the DESeq2 normalization method. Both treatment (siRNA VINK versus siRNA Ctrl) and batch (replicate) effects were taken into account to estimate parameters and fit the model, in order to apply the Wald test. Bigwig files were generated using rtracklayer Bioconductor-R package, version 3.10, with 1-bp binning and normalization based on the total number of reads aligned. Spliced reads of VINK were obtained by using the GenomicAlignments library (version 1.22.1) in R script and the human reference genome (BSgenome.Hsapiens.UCSC.hg38, version 1.4.1). Mapped paired-end reads were read from BAM with the “readGAlignmentPairs” function. By using “njunc” and “junction” functions, the number of spliced reads and genomic coordinates of junctions were obtained (53). Spliced junctions were analyzed as described elsewhere (16). The four siCtle samples from the RNA-Seq shown in Fig. 1D were combined and analyzed for the detection of spliced junctions. For each detected junction, we calculated the number of reads which contained it. Table 1 shows spliced junctions detected on more than 10 reads.

### Data availability.

RNA-Seq data are available in the Geo database under accession number GSE197308.
